# Association between Scabies Treatment and Parkinson’s Disease: A Nationwide, Population-Based Study

**DOI:** 10.3390/ph17101342

**Published:** 2024-10-08

**Authors:** Kao-Sung Tsai, Ming-Kuei Lu, Chao-Hong Liu, Fuu-Jen Tsai, Wen-Chi Chen, Huey-Yi Chen, Heng-Jun Lin, Cheng-Li Lin, Jen-Chih Lee, Kee-Ming Man, Chien-Yi Ho, Yung-Hsiang Chen

**Affiliations:** 1 School of Chinese Medicine, Graduate Institute of Integrated Medicine, College of Chinese Medicine, Ph.D. Program for Translational Medicine, College of Medicine, China Medical University, Taichung 404, Taiwan; raysclinic@gmail.com (K.-S.T.); mklu@cmu.edu.tw (M.-K.L.); 000704@tool.caaumed.org.tw (F.-J.T.); wgchen@mail.cmu.edu.tw (W.-C.C.); d888208@ms45.hinet.net (H.-Y.C.); t38958@mail.cmuh.org.tw (H.-J.L.); orangechengli@gmail.com (C.-L.L.); d10139@mail.cmuhch.org.tw (C.-Y.H.); 2 Million-Person Precision Medicine Initiative, Departments of Dermatology, Neurology, Medical Genetics, Obstetrics and Gynecology, Urology, and Medical Research, Management Office for Health Data, China Medical University Hospital, Taichung 404, Taiwan; 3 Department of Applied Cosmetology, Hungkuang University, Taichung 433, Taiwan; 4 Department of Electronics Engineering, National Kaohsiung University of Science and Technology, Kaohsiung 824, Taiwan; y4509@yuanhosp.com.tw; 5 Department of Dermatology, Yuan’s General Hospital, Kaohsiung 802, Taiwan; 6 Division of Medical Genetics, China Medical University Children’s Hospital, Taichung 404, Taiwan; 7 Departments of Pulmonary and Critical Care Medicine, Anesthesiology, Medical Research, Division of Family Medicine, Physical Examination Center, China Medical University Hsinchu Hospital, Hsinchu 302, Taiwan; man_jimmy60@hotmail.com; 8 Department of Nursing, College of Medical Technology and Nursing, Yuanpei University of Medical Technology, Hsinchu 300, Taiwan; 9 Department of Psychology, College of Medical and Health Science, Asia University, Taichung 413, Taiwan

**Keywords:** scabies, Parkinson’s disease, National Health Insurance Research Database, lindane

## Abstract

Background: Scabies is typically treated with scabicides like lindane, which poses a risk for acute neural toxicity. Lindane’s prolonged use, particularly in agriculture, is linked to neurodegenerative diseases, including Parkinson’s disease (PD), the second most common neurodegenerative disorder. This study aimed to evaluate whether scabies patients, particularly those treated with topical lindane, are at increased risk of developing PD. Methods: A nationwide population-based cohort study was conducted using data from Taiwan’s National Health Research Institutes claims database from 2000 to 2018. The study included 27,173 patients with scabies, matched to a control group, with both groups followed for up to 18 years. The primary outcome was the incidence of newly diagnosed PD, and the hazard ratio (HR) for PD was calculated, focusing on those treated with topical lindane. Results: Among the 54,346 patients, 1639 (3.0%) were newly diagnosed with PD, with 993 (60.6%) from the scabies group and 646 (39.4%) from the control group. Scabies patients had an adjusted hazard ratio (aHR) of 1.46 (95% CI 1.32–1.63) for developing PD compared to controls. However, patients treated with topical lindane had a significantly lower aHR for PD at 0.15 (95% CI 0.12–0.19; *p* < 0.001), with a lower cumulative incidence of PD also observed in this group (*p* < 0.001). Conclusions: Scabies patients are at a 1.46-fold increased risk of developing PD, but those treated with lindane exhibit a significantly lower risk, suggesting potential protective effects of lindane against PD.

## 1. Introduction

Parkinson’s disease (PD) is the second most common neurodegenerative disease, exhibiting diverse clinical features, leading to decreased quality of life, disability, and death among the elderly [1,2]. Pathological hallmarks of PD include the selective loss of dopaminergic neurons originating from the substantia nigra and the accumulation of neuronal α-synuclein immunoreactive inclusions in glial cells. The etiology of PD has yet to be clearly elucidated; however, genetic, intrinsic inflammation, and environmental factors play a prominent role in disease occurrence. Despite various ecological factors, pesticide exposure is considered to be the primary factor in the pathogenesis of PD [3]; however, some evidence suggests that central and peripheral inflammation also play vital roles. Several peripheral biomarkers are used to accurately trace and detect disease severity and progression [4,5,6,7].

Scabies infestation is a common contagious parasitic disease of the skin caused by *Sarcoptes scabiei.* The annual global incidence of scabies is approximately 300 million cases and shows an upward trend every year. In 2021, approximately 622 million incident cases of scabies occurred globally, which places a heavy burden on infected individuals and healthcare systems [8,9]. Most patients with scabies present with pruritus, particularly at night, which often causes sleep problems, thus severely impacting the quality of life of those affected [10,11]. Previous studies have identified scabies-related complications and an increased risk for stroke, acute myocardial infarction, chronic obstructive pulmonary disease (COPD), chronic kidney disease, bipolar disorder, and intellectual disability in children [12,13,14,15,16,17]. Although the association between scabies infestation and these diseases remains unclear, immune and inflammatory responses play a vital role in the systemic impact of scabies [18]. Lindane is an effective insecticide commonly used for the treatment of scabies [19]. However, owing to its neurotoxic effects, lindane has been limited in its concentration, dosage, and frequency in clinical use; as such, since 1995, it has been used in the United States only as a second-line therapy. Based on the immune inflammatory response to scabies infection and the neurotoxicity caused by first-line use, lindane could increase the risk for neural degeneration. Prompted by the lack of previous studies investigating the relationship between scabies and PD, this study aimed to assess various risks for PD in patients with scabies treated with or without topical lindane using information from a nationwide database. This is aimed to be part of a structured initiative to establish evidence-based clinical recommendations for managing comorbidities in scabies.

## 2. Results

### 2.1. Baseline Characteristics of Subjects and Comorbidities

The present study included data from 27,173 patients with and without scabies. Demographic characteristics of the study and control groups are summarized in Table 1. The mean ages of the scabies and non-scabies cohorts were 54.55 and 54.51 years, respectively, with approximately 43.32% of patients < 50 years of age and males outnumbering females. Frequency matching according to age and sex with the case group resulted in no significant differences between the 2 groups for these variables. Patients in the scabies cohort were more likely to experience cerebrovascular accident (CVA) (*p* < 0.001), hypertension (HTN) (*p* < 0.001), diabetes mellitus (DM) (*p* < 0.001), COPD (*p* < 0.001), coronary heart disease (*p* < 0.001), dementia (*p* < 0.001), Alzheimer’s disease (AD)/Lewy body dementia (*p* < 0.001), chronic kidney disease (*p* < 0.001), anxiety/depressive disorder (*p* < 0.001), and sleep disorder(s) (*p* < 0.001) than those in the non-scabies cohort. The mean follow-up for the scabies and non-scabies cohorts was 7.96 and 8.54 years, respectively.

### 2.2. Risk for PD Associated with Scabies Infection

**Figure 1:**
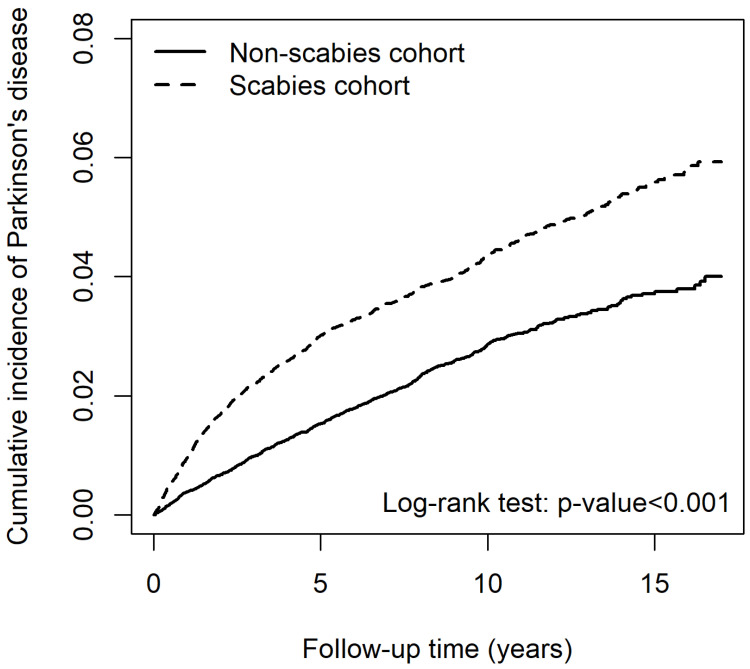
Cumulative incidence of Parkinson’s disease between patients with and without scabies.

Patients with scabies had a significantly greater risk for developing PD than those without scabies (adjusted HR [aHR] 1.46 [95% CI 1.32–1.63]) (Table 2). Results of log-rank analysis revealed that the risk for PD increased in patients with scabies infection (Figure 1). After adjusting for age, sex, and comorbidities, other risk factors for PD included older age, male sex (aHR 1.15 [95% CI 1.04–1.27]), with CVA (aHR 1.53 [95% CI 1.36–1.71), HTN (aHR 1.28 [95% CI 1.12–1.46]), COPD (aHR 1.17 [95% CI 1.05–1.31]), dementia (aHR 1.38 [95% CI 1.19–1.61]), AD/Lewy body dementia (aHR 1.33 [95% CI 1.05–1.69]), anxiety/depressive disorder (aHR 1.26 [95% CI 1.13–1.41]) and sleep disorder(s) (aHR 1.19 [95% CI 1.06–1.33]). The cumulative incidence of PD in patients with scabies was significantly higher than that in those without scabies (*p* < 0.001) as the follow-up increased (Figure 1).

**Figure 2:**
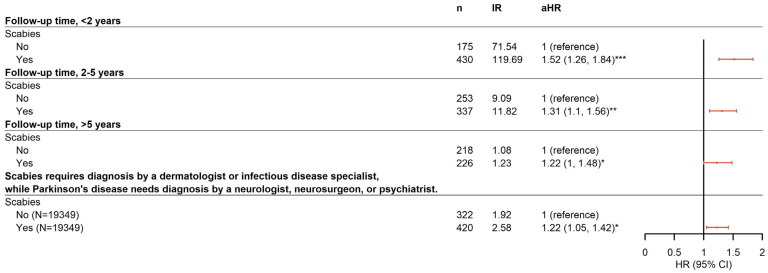
Forest plot for the sensitivity analysis. * *p* < 0.05, ** *p* < 0.01, *** *p* < 0.001.

Furthermore, a stratified analysis was performed to determine whether the duration of scabies infection was associated with the risk for PD. Data reported in Figure 2 indicate that the risk for PD occurring within 2 years after infection with scabies (aHR 1.52 [95% CI 1.26–1.84]) was slightly higher than that between 2 and 5 years (aHR 1.31 [95% CI 1.10–1.56]) and >5 years (aHR 1.22 [95% CI 1.00–1.48]) after adjusting for age, sex, and comorbidities.

### 2.3. Baseline Characteristics of Subjects Treated for Scabies and Comorbidities

The baseline characteristics of the study population in Study II are summarized in Table 3. Study participants comprised 8457 individuals who did not use scabies medication (control cohort) and 8457 who did (case–cohort). Lindane was the most frequently prescribed medication (99.31%). Only 27 (0.32%) patients were prescribed permethrin alone, and 31 (0.37%) used a combination of permethrin and lindane. No oral ivermectin prescriptions were recorded in this study. The proportion of male subjects in both the case and control groups was >50% (59.06%). The mean age was 61.11 and 61.13 years with and without scabies medication, respectively. A comparison of comorbidities, including CVA, HTN, DM, COPD, dementia, AD/Lewy body dementia, anxiety/depressive disorder, and sleep disorder(s), exhibited significant differences in distribution (all *p* < 0.001).

### 2.4. Risk for PD-Associated Lindane Use in Patients with Scabies

**Figure 3:**
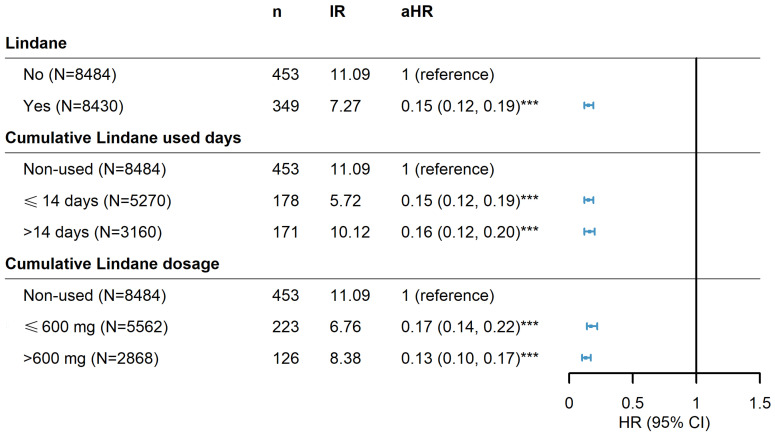
Hazard ratios and 95% confidence intervals of Parkinson’s disease risk associated with cumulative doses and drug-use days of lindane among scabies patients. *** *p* < 0.001.

Risks for PD associated with lindane use are summarized in Table 4. Lindane medication users had a significantly lower risk for PD than non-lindane patients (aHR 0.15 [95% CI 0.12–0.19]) after controlling for age, sex, and comorbidities. The log-rank test also revealed that the risk for PD was reduced in lindane users (Figure 3). The age group 50 to 69 years (aHR 3.31 [95% CI 2.55–4.30]) and >70 years (aHR 5.90 [95% CI 4.56–7.63]) exhibited a significantly higher risk for PD than the 20–49 years’ age group. Patients with CVA (aHR 1.84 [95% CI 1.45–2.34]), HTN (aHR 2.32 [95% CI 1.72–3.13]), COPD (aHR 1.36 [95% CI 1.10–1.69]), dementia (aHR 1.48 [95% CI 1.19–1.84]), anxiety/depressive disorder (aHR 1.37 [95% CI 1.11–1.68]), and sleep disorder(s) (aHR 1.36 [95% CI 1.11–1.67]) exhibited a specific increased risk for PD than without these comorbidities.

In addition, a stratified analysis was performed to determine whether prescription duration or dosage of lindane affected the risk for PD. Fourteen days of lindane (600 mg), which is equal to a 1% concentration of 30 g once per week for 2 consecutive weeks in a common clinical prescription, was evaluated. Results indicated that the duration or cumulative dose of lindane did not impact the trends in the risk of developing Parkinson’s disease (Figure 3).

**Figure 4:**
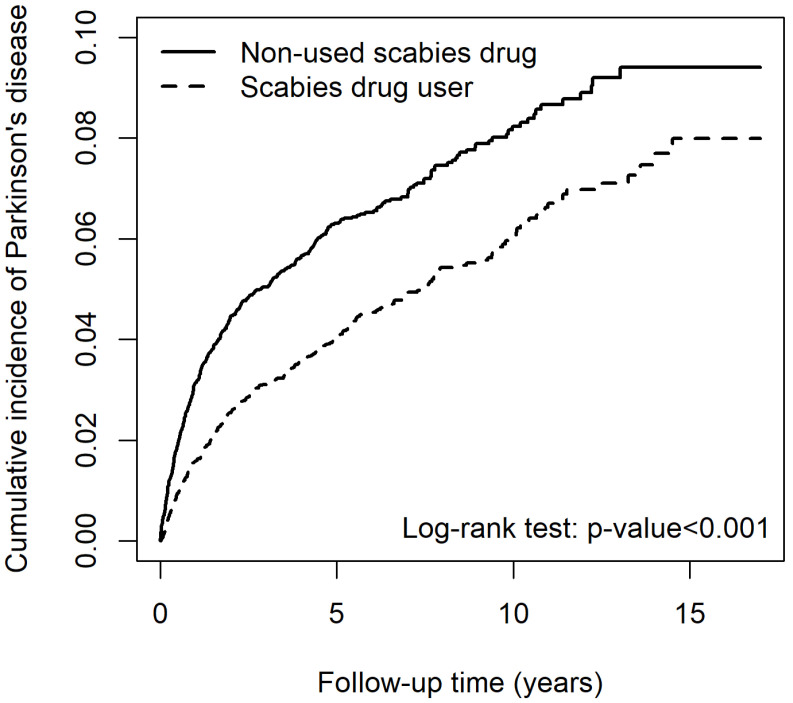
Cumulative incidence of Parkinson’s disease for scabies cohort between patients that used and did not use lindane.

Patients among the scabies cohort undergoing lindane treatment exhibited a significantly lower cumulative incidence of PD than those who were not (*p* < 0.001) (Figure 4).

## 3. Discussion

To our knowledge, this is the first population-based cohort study to investigate the association between scabies and PD. Results of this study demonstrated that patients with scabies have an increased risk for developing PD. However, among the scabies-infected groups, patients treated with lindane exhibited a reduced risk for PD compared with those not treated with lindane. Awareness of this finding may help clinicians remain alert to any signs or symptoms of PD in patients with previous scabies infections.

Immune and inflammatory responses are the basic pathophysiological mechanisms underlying scabies. The immune response against common scabies is dominated by T-helper (Th) 1 cells and their cytokine profiles, whereas the response against crusted scabies is dominated by immunoglobulin E-driven Th2 cells [20]. According to previous studies, pro-inflammatory cytokines, including interleukin (IL)-1, IL-6, IL-8, and tumor necrosis factor-alpha (TNF-α), as well as the immunomodulatory cytokines IL-10 and IL-12, are responsible for the immune response to scabies infections [21]. IL-6 also plays a key role in the development of inflammatory diseases. It is a pro-inflammatory cytokine that signals through the JAK/STAT pathway and is associated with many chronic inflammatory diseases. IL-6 promotes the pro-inflammatory functions of Th17 and M1 macrophages while promoting anti-inflammatory Th2 differentiation and cytokine secretion. IL-6 also stimulates profibrotic responses in M2 macrophages, in conjunction with M1/M2 polarization sustained by the Th2 cytokines IL-4 and IL-23. Transforming growth factor-beta contributes to IL-6-dependent differentiation of Th17, essential factors leading to many inflammatory diseases [22,23,24]. On the other hand, there were significant increases in inflammatory cytokine levels (i.e., IL-6, IL-1β, and TNF-α) in both peripheral blood and cerebrospinal fluid among patients with PD compared with healthy controls [22]. For decades, the blood–brain barrier has been widely believed to provide an immune-privileged environment in the central nervous system (CNS) by blocking peripheral immune cells and humoral immune factors. However, there is increasing evidence that the peripheral immune system plays a critical role in regulating CNS homeostasis and disease [25]. Peripherally released cytokines can cross the blood–brain barrier and cause direct neurotoxicity, and contribute to the activation of microglia and astrocytes, which contribute to the progression of neuroinflammatory and neurodegenerative diseases [26,27]. However, inconsistent results for these markers in the CNS and peripheral blood of patients with scabies infestation urgently need to be explored in future studies.

The cumulative incidence of PD between patients with and without scabies exhibited an excess risk for PD in the scabies group, occurring in the initial 5-year follow-up (Figure 1). Lysen et al. reported that poor sleep quality and short duration increased the risk for PD, especially during the first 2 years of follow-up [28]. Therefore, we believe that, in addition to skin inflammation in scabies dermatitis, pruritus caused by scabies, which interferes with sleep, is an extremely important symptom. Several studies have demonstrated that insomnia is associated with further development of PD [29]. Sohail et al. reported that increased actigraphy-derived sleep fragmentation in older subjects without PD was associated with an increased burden of PD pathology on brain autopsy, indicating that objective disturbances―aside from subjectively impaired sleep―are related to PD pathology [30]. Although the mechanisms underlying insomnia and the risk for PD are unclear, the effect of sleep disturbance on neurodegenerative diseases is plausible because sleep deprivation promotes oxidative stress. Furthermore, elevated levels of reactive oxygen species (ROS) leading to subsequent mitochondrial dysfunction, disturbances in cellular and energy balance, compromised synaptic activity and neurotransmission in neurons, or reduced clearance of metabolic waste, including extracellular alpha-synuclein, all correlate with cognitive decline linked to PD [31].

Several anti-scabietics treatment options are available, including topically applied permethrin, lindane, benzyl benzoate (BB), sulfur, malathion, and crotamiton, as well as oral ivermectin. In the United States, three topical treatments are approved by the U.S. Food and Drug Administration (FDA) for the management of scabies in adults: permethrin 5% cream, crotamiton 10% cream or lotion, and Spinosad 0.9% topical suspension. The 2017 European guidelines for the management of scabies currently recommend repeated administration of topical permethrin 5%, topical BB 10–25%, or systemic ivermectin at a dose of 200 µg/kg body weight as first-line treatments [9]. The treatment protocol for scabies has evolved over time and will likely continue to develop, particularly as the *Sarcoptes scabiei* mite exhibits increasing resistance to commonly prescribed insecticides [32,33]. Additionally, the availability of scabicides and prescription preferences may vary between countries [34].

Topical lindane, also known as gamma-hexachlorocyclohexane (γ-HCH) or gamma-benzene hexachloride (γ-BHC), was previously accepted as the standard of care for most cases of scabies and lice before evidence of central neurotoxicity was revealed [35]. In addition to pharmaceutical treatments, lindane is an organochlorine chemical and an isomer of hexachlorocyclohexane, which has been used as an agricultural insecticide [36]. Accumulating evidence suggests that environmental factors, such as pesticide exposure, are involved in the etiology of PD, and elevated levels of organochlorine pesticides have been found in the substantia nigra of patients with PD [37,38]. In the present study, subjects with scabies treated with topical lindane exhibited a decreased incidence of PD compared with the lindane treatment group because the physician was aware of the risk for neural toxicity of lindane and restricted its concentration and dosage in clinical practice. Furthermore, the clinical use of topical lindane differs from the long-term accumulation of pesticides in the environment or food contamination mainly absorbed through the gut [39,40,41].

The present study had some limitations. In many countries, lindane is no longer approved, and the primary treatment of choice is a combination of orally administered ivermectin and local permethrin. However, topical permethrin was issued by the Taiwan Food and Drug Agency and covered by Taiwan’s health insurance in September 2017, whereas oral ivermectin was not issued until August 2018. This study analyzed all subjects and related drug use up to December 2018. The number of subjects was insufficient, and there was no statistically significant difference in the results. Comparison of these medicines with lindane and the risk for PD is also needed for further evaluation. Second, some potential risk factors, including subjects’ occupation, place of residence, and whether the residents were in a nursing home or hospitalized, were not included in our analyses because these data were not available. Third, the severity of PD and scabies infection could not be evaluated. Fourth, the cross-sectional design of the study precluded the direct determination of a causal relationship, if any, between scabies, topical lindane, and PD [42,43,44,45].

## 4. Materials and Methods

### 4.1. Data Source and Collection

This study is a population-based retrospective cohort study using data from Taiwan’s National Health Insurance Research Database (NHIRD). Taiwan’s National Health Insurance (NHI) program, implemented by the government in March 1995, provided comprehensive healthcare to almost all Taiwanese citizens, with a coverage rate of more than 99% of Taiwan’s entire population, and contracted with 97% of hospitals and 92% of clinics. The National Health Research Institute (NHRI) of Taiwan manages and publicly releases for research purposes multiple NHI databases that include information about essential patient characteristics, date of visit, diagnosis codes for the International Classification of Diseases, Ninth Revision, Clinical Modification (ICD-9-CM and ICD-10-CM) codes, detailed claims data for examinations, disease management, and drug prescriptions for all admitted patients and outpatients [46]. This study used a sub-dataset of the NHIRD, which is called the Longitudinal Generation Tracking Database 2000 (LGTD 2000). The LGTD 2000 database contains data for 2 million people randomly selected from the NHIRD in 2000. The LGTD 2000 and NHIRD are similar regarding demographic data and origin population. This study was approved by the Institutional Review Board of CMUH111-REC2-109(CR-1) with the institutional review board approval of the Public Health, Social, and Behavioral Science Committee Research Ethics Committee, China Medical University and Hospital.

### 4.2. Study Design and Population

**Figure 5:**
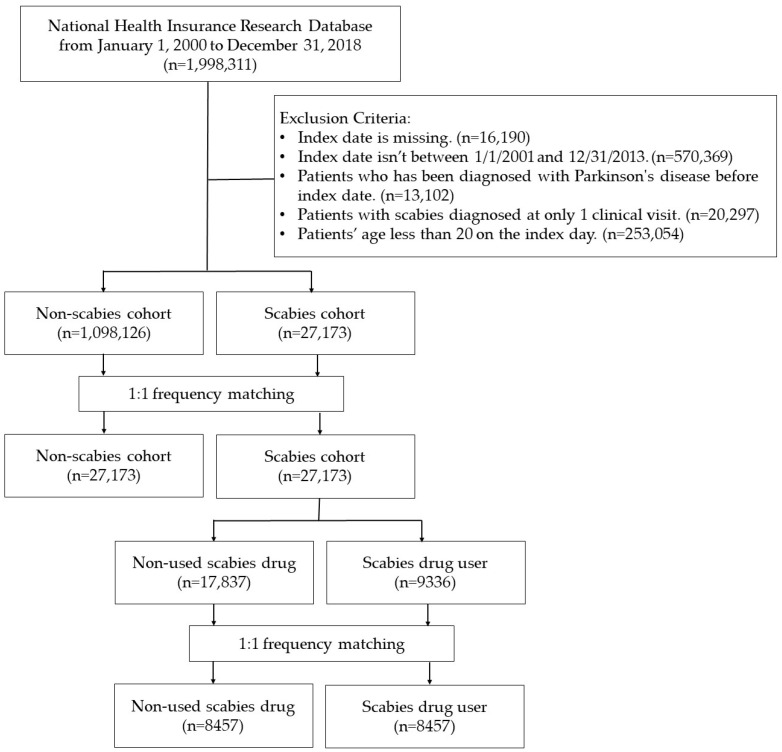
Flowchart.

Two retrospective cohort studies utilizing a nationwide database were conducted in this research. The flowchart of the study is presented in Figure 5. In Study I, patients over 20 years old with scabies (ICD-9-CM: 133.0; ICD-10-CM: B86) diagnosed with at least two clinical visits or one hospital admission between 1 January 2001, and 31 December 2013, were designated as the scabies cohort. These patients were newly diagnosed with PD (ICD-9-CM: 332; ICD-10-CM: G20, G21) or followed up until December 31, 2018. Subjects with a history of PD before the enrollment date were excluded from the study group. For the patients in the study group, each patient’s date of diagnosis of scabies was assigned as the index date and considered the starting point for their investigation. For the control group, we randomly selected index dates between 2001 and 2017. Sensitivity analyses were conducted, requiring a dermatologist or infectious disease specialist to evaluate scabies diagnoses. For PD diagnoses, a neurologist, neurosurgeon, or psychiatrist’s evaluation was essential. These results are shown in Figure 5.

In Study II, coding for scabies drugs was obtained for medication variant control in advanced analysis steps. Treatment was divided into scabies drug users and non-scabies drug users. The date of initial accepted drug treatment after the new diagnosis of scabies was used as the index date for the cohort group. All subjects were followed from the index date to the occurrence of the endpoint or until 31 December 2018, whichever came first, with observations on the last dates considered censored.

### 4.3. Comparison Group

Subjects without scabies were randomly selected from the same dataset. Each patient with newly diagnosed scabies in the NHRI database was pair-matched with one issue of the same age (at every 5 years), sex, and index year. Topical lindane medications and comorbidities were not matched. We selected comparison subjects using incidence density sampling by computer programming [47]. In the comparison group, subjects who had a history of PD before enrollment were also excluded from the study group. To determine PD and survival analyses adjusting for age, sex, comorbidities, and medications were carried out with Cox’s proportional hazards model. All enrollees were followed from the date of enrollment until the first diagnosis of PD or censored date of death, withdrawal from the insurance, or until 31 December 2018.

### 4.4. Potential Confounders

In analyzing the effect of risk and different treatments in patients with scabies on the outcome of PD, we controlled for age and sex. We identified the following comorbidities as covariates for this study: Cerebrovascular accident (CVA, ICD-9-CM: 430–438, ICD-10-CM: I60–I69), hypertension (HTN, ICD-9-CM: 401–405, ICD-10-CM: I10–I15), diabetes (DM, ICD-9-CM: 250, ICD-10-CM: E08–E31), chronic obstructive pulmonary disease (COPD, ICD-9-CM: 491, 492, and 496, ICD-10-CM: J41–J44), coronary heart disease (CHD, ICD-9-CM: 410–414; ICD-10-CM: I20–I25), dementia (ICD-9-CM:290 and 294.1, ICD-10-CM: F01-F03 and F05), Alzheimer’s disease /Lewy body dementia (ICD-9-CM: 330.8, 330.9, 331.0, 331.1, 331.2, 331.7, 331.8, 331.9, 334.4, 336.2 and 349.89, ICD-10-CM: G30-G32 and G93.7), chronic kidney disease (CKD; ICD-9-CM: 585, 586 and 588, ICD-10-CM: N18, N19, N03, and N04), anxiety/depressive disorder (ICD-9-CM: 300 and 311, ICD-10-CM: F30–F48), and sleep disorder (ICD-9-CM: 307.4, 780.5, 780.51, 780.52, 780.53, 780.57, and 780.59, ICD-10-CM: G47.0, G47.2, G47.3, G47.8, G47.9, and F51.0).

### 4.5. Statistical Analyses

Person years of two populations were calculated from baseline to the occurrence of PD or closing date (31 December 2018). All statistical analyses were performed using SAS version 9.4 software (SAS Institute, Inc., Cary, NC, USA). All data are expressed as means, standard deviations, or *n* (%) unless otherwise stated. Comparisons between groups were performed using Student’s *t*-test for continuous variables and Pearson’s chi-square test, as appropriate, for categorical variables. The Cox’s proportional hazards model was used to estimate the hazard ratio for the progression of the outcome. The Cox proportional hazard model was used to calculate the hazard ratios and 95% confidence interval of PD for patients with scabies compared with non-scabies subjects. To verify that the data meet the PH assumption, we used Cox–Snell residuals. A plot of the estimated cumulative hazards function against the Cox–Snell residuals was generated, and we confirmed that it closely follows a 45-degree line, indicating that the proportional hazards assumption holds for our model. All the models were adjusted for the variables (gender, age, and comorbidities). The Kaplan–Meier method was used to plot the cumulative incidence. Individuals with missing data were excluded from the analyses to ensure data integrity. Baseline characteristics between the experimental and control groups were assessed using the *t*-test for continuous variables and the Chi-square test for categorical variables to ensure balance between the groups. The Kaplan–Meier method was used to plot the cumulative incidence curve for Parkinson’s disease. To estimate the risk, we applied univariate and multivariate Cox proportional hazards models to calculate HRs and 95% CIs for Parkinson’s disease in scabies patients compared with non-scabies subjects. The multivariate Cox model was adjusted for key covariates, including sex, age, and comorbidities, to control for potential confounding factors. All statistical tests were performed at the two-tailed significance level of 0.05. A *p*-value < 0.05 was considered statistically significant.

## 5. Conclusions

Results of the present study indicated that patients with scabies are at an increased risk for developing PD. Furthermore, patients with scabies treated with topical lindane exhibited a lower incidence of PD than non-infected individuals treated with lindane. These findings suggest that early and aggressive treatment of scabies may lower the risk for subsequent development of PD.

## Figures and Tables

**Table 1:** Baseline characteristics for the scabies cohort and comparison cohorts.

Variables	Scabies				*p*-Value
	No (*n* = 27,173)		Yes (*n* = 27,173)		
	*n*	%	*n*	%	
Age, years					>0.999
20–49	11,770	43.32	11,770	43.32	
50–69	7009	25.79	7009	25.79	
≥70	8394	30.89	8394	30.89	
Mean ± SD ^a^	54.51	20.84	54.55	20.92	0.823
Sex					>0.999
Female	11,732	43.18	11,732	43.18	
Male	15,441	56.82	15,441	56.82	
Comorbidities					
Cerebrovascular accident	3404	12.53	7423	27.32	<0.001
Hypertension	9544	35.12	12,328	45.37	<0.001
Diabetes	4472	16.46	6977	25.68	<0.001
Chronic obstructive pulmonary disease	4549	16.74	7372	27.13	<0.001
Coronary heart disease	4971	18.29	6823	25.11	<0.001
Dementia	809	2.98	2917	10.73	<0.001
Alzheimer’s disease/Lewy body dementia	300	1.10	651	2.40	<0.001
Chronic kidney disease	1078	3.97	2097	7.72	<0.001
Anxiety/Depressive disorder	5314	19.56	7529	27.71	<0.001
Sleep disorders	5881	21.64	8380	30.84	<0.001
Follow-up time, years					
Mean ± SD ^a^	8.54	4.59	7.96	4.89	<0.001

^a^ *t*-test; Chi-square test; SD: standard deviation.

**Table 2:** Cox model measured hazard ratio and 95% confidence intervals of Parkinson’s disease-associated scabies in patients.

Variables	Parkinson’s Disease			cHR	(95% CI)	*p*-Value	aHR	(95% CI)	*p*-Value
	*n*	PY	IR						
Scabies									
No	646	232,173.69	2.78	1.00	(reference)	-	1.00	(reference)	-
Yes	993	216,256.68	4.59	1.64	(1.48, 1.81) ***	<0.001	1.46	(1.32, 1.63) ***	<0.001
Age, years									
20–49	156	245,166.57	0.64	1.00	(reference)	-	1.00	(reference)	-
50–69	452	119,717.56	3.78	5.90	(4.92, 7.08) ***	<0.001	4.46	(3.68, 5.41) ***	<0.001
≥70	1031	83,546.24	12.34	18.50	(15.61, 21.97) ***	<0.001	10.49	(8.59, 12.82) ***	<0.001
Sex									
Female	738	201,356.80	3.67	1.00	(reference)	-	1.00	(reference)	-
Male	901	247,073.58	3.65	0.98	(0.89, 1.08)	0.648	1.15	(1.04, 1.27) **	0.005
Comorbidities									
CVA									
No	940	395,558.29	2.38	1.00	(reference)	-	1.00	(reference)	-
Yes	699	52,872.09	13.22	5.07	(4.58, 5.6) ***	<0.001	1.53	(1.36, 1.71) ***	<0.001
Hypertension									
No	494	315,302.97	1.57	1.00	(reference)	-	1.00	(reference)	-
Yes	1145	133,127.41	8.60	5.14	(4.62, 5.72) ***	<0.001	1.28	(1.12, 1.46) ***	<0.001
Diabetes									
No	1058	381,136.81	2.78	1.00	(reference)	-	1.00	(reference)	-
Yes	581	67,293.57	8.63	2.86	(2.59, 3.17) ***	<0.001	1.11	(1, 1.24)	0.057
COPD									
No	980	380,942.81	2.57	1.00	(reference)	-	1.00	(reference)	-
Yes	659	67,487.57	9.76	3.50	(3.16, 3.86) ***	<0.001	1.17	(1.05, 1.31) **	0.005
CHD									
No	958	381,549.46	2.51	1.00	(reference)	-	1.00	(reference)	-
Yes	681	66,880.92	10.18	3.73	(3.38, 4.13) ***	<0.001	1.06	(0.94, 1.18)	0.337
Dementia									
No	1361	435,390.77	3.13	1.00	(reference)	-	1.00	(reference)	-
Yes	278	13,039.61	21.32	5.73	(5.02, 6.54) ***	<0.001	1.38	(1.19, 1.61) ***	<0.001
AD/Lewy body dementia									
No	1563	444,024.68	3.52	1.00	(reference)	-	1.00	(reference)	-
Yes	76	4405.70	17.25	4.29	(3.41, 5.4) ***	<0.001	1.33	(1.05, 1.69) *	0.02
CKD									
No	1504	434,047.31	3.47	1.00	(reference)	-	1.00	(reference)	-
Yes	135	14,383.07	9.39	2.35	(1.97, 2.81) ***	<0.001	0.88	(0.73, 1.05)	0.163
Anxiety/Depressive disorder									
No	975	357,061.40	2.73	1.00	(reference)	-	1.00	(reference)	-
Yes	664	91,368.97	7.27	2.54	(2.3, 2.8) ***	<0.001	1.26	(1.13, 1.41) ***	<0.001
Sleep disorders									
No	963	352,130.28	2.73	1.00	(reference)	-	1.00	(reference)	-
Yes	676	96,300.10	7.02	2.41	(2.18, 2.66) ***	<0.001	1.19	(1.06, 1.33) **	0.002

PY: person-year; IR: incidence rate, per 1000 person-years; cHR: crude hazard ratio; aHR: adjusted hazard ratio, adjusted for age, sex, and comorbidities; CI: confidence interval; * *p* < 0.05, ** *p* < 0.01, *** *p* < 0.001.

**Table 3:** Baseline characteristics for scabies medications used cohort and comparison cohorts.

Variables	Scabies Drug Used				*p*-Value
	No (*n* = 8457)		Yes (*n* = 8457)		
	*n*	%	*n*	%	
Scabies drug					
Lindane	-	-	8399	99.31	
Permethrin	-	-	27	0.32	
Lindane + Permethrin	-	-	31	0.37	
Age, years					>0.999
20–49	2592	30.65	2592	30.65	
50–69	2275	26.90	2275	26.90	
≥70	3590	42.45	3590	42.45	
mean ± SD ^a^	61.13	20.14	61.11	20.22	0.959
Sex					>0.999
Female	3533	41.78	3533	41.78	
Male	4924	58.22	4924	58.22	
Comorbidities					
CVA	204	2.41	3430	40.56	<0.001
Hypertension	258	3.05	5005	59.18	<0.001
Diabetes	143	1.69	2895	34.23	<0.001
COPD	195	2.31	3366	39.80	<0.001
Coronary heart disease	171	2.02	2879	34.04	<0.001
Dementia	107	1.27	1571	18.58	<0.001
AD/Lewy body dementia	28	0.33	319	3.77	<0.001
CKD	58	0.69	954	11.28	<0.001
Anxiety/Depressive disorder	146	1.73	2952	34.91	<0.001
Sleep disorders	149	1.76	3386	40.04	<0.001
Follow-up time, years					
mean ± SD ^a^	4.83	4.27	5.68	4.29	<0.001

^a^ *t*-test; Chi-square test; SD: standard deviation.

**Table 4:** Cox model measured hazard ratio and 95% confidence intervals of Parkinson’s disease-associated used Scabies drug in patients with scabies.

Variables	Parkinson’s Disease			cHR	(95% CI)	*p*-Value	aHR	(95% CI)	*p*-Value
	*n*	PY	IR						
Scabies drugs									
No	453	40,839.15	11.09	1.00	(reference)	-	1.00	(reference)	-
Yes	349	48,020.43	7.27	0.68	(0.59, 0.78) ***	<0.001	0.15	(0.12, 0.19) ***	<0.001
Age, years									
20–49	79	41,582.54	1.90	1.00	(reference)	-	1.00	(reference)	-
50–69	227	26,759.53	8.48	4.17	(3.22, 5.38) ***	<0.001	3.31	(2.55, 4.3) ***	<0.001
≥70	496	20,517.51	24.17	9.70	(7.61, 12.36) ***	<0.001	5.90	(4.56, 7.63) ***	<0.001
Sex									
Female	368	38,791.69	9.49	1.00	(reference)	-	1.00	(reference)	-
Male	434	50,067.89	8.67	0.88	(0.77, 1.01)	0.076	1.03	(0.89, 1.18)	0.708
Comorbidities									
CVA									
No	500	75,868.65	6.59	1.00	(reference)	-	1.00	(reference)	-
Yes	302	12,990.93	23.25	2.90	(2.51, 3.35) ***	<0.001	1.84	(1.45, 2.34) ***	<0.001
Hypertension									
No	427	66,920.55	6.38	1.00	(reference)	-	1.00	(reference)	-
Yes	375	21,939.03	17.09	2.32	(2.02, 2.67) ***	<0.001	2.32	(1.72, 3.13) ***	<0.001
Diabetes									
No	604	76,695.44	7.88	1.00	(reference)	-	1.00	(reference)	-
Yes	198	12,164.13	16.28	1.78	(1.52, 2.09) ***	<0.001	1.09	(0.89, 1.33)	0.418
COPD									
No	532	74,984.21	7.09	1.00	(reference)	-	1.00	(reference)	-
Yes	270	13,875.37	19.46	2.36	(2.03, 2.73) ***	<0.001	1.36	(1.1, 1.69) **	0.005
CHD									
No	586	77,127.12	7.60	1.00	(reference)	-	1.00	(reference)	-
Yes	216	11,732.46	18.41	2.06	(1.76, 2.41) ***	<0.001	0.85	(0.69, 1.05)	0.129
Dementia									
No	632	84,315.75	7.50	1.00	(reference)	-	1.00	(reference)	-
Yes	170	4543.83	37.41	3.68	(3.1, 4.37) ***	<0.001	1.48	(1.19, 1.84) ***	<0.001
AD/Lewy body dementia									
No	758	87,734.30	8.64	1.00	(reference)	-	1.00	(reference)	-
Yes	44	1125.28	39.10	3.56	(2.63, 4.83) ***	<0.001	1.50	(1.08, 2.07) *	0.015
CKD									
No	748	85,480.57	8.75	1.00	(reference)	-	1.00	(reference)	-
Yes	54	3379.01	15.98	1.47	(1.12, 1.94) **	0.006	0.72	(0.54, 0.96) *	0.027
Anxiety/Depressive disorder									
No	575	73,425.78	7.83	1.00	(reference)	-	1.00	(reference)	-
Yes	227	15,433.80	14.71	1.79	(1.53, 2.08) ***	<0.001	1.37	(1.11, 1.68) **	0.003
Sleep disorders									
No	559	71,771.41	7.79	1.00	(reference)	-	1.00	(reference)	-
Yes	243	17,088.16	14.22	1.71	(1.47, 1.98) ***	<0.001	1.36	(1.11, 1.67) **	0.003

PY: person-year; IR: incidence rate, per 1000 person-years; cHR: crude hazard ratio; aHR: adjusted hazard ratio, adjusted for age, sex, and comorbidities; CI: confidence interval; * *p* < 0.05, ** *p* < 0.01, *** *p* < 0.001.

## Data Availability

The original contributions presented in the study are included in the article; further inquiries can be directed to the corresponding author.

## Abbreviations

PD: Parkinson’s disease; cHR, crude hazard ratio; aHR, adjusted hazard ratio; DA, dopaminergic; SN, substantia nigra; α-syn, α-synuclein; CVA, cerebrovascular accident; HTN, hypertension, DM, diabetes mellitus; AD, Alzheimer’s disease; CKD, chronic kidney disease.
